# Versatile Liquid Metal/Alginate Composite Fibers with Enhanced Flame Retardancy and Triboelectric Performance for Smart Wearable Textiles

**DOI:** 10.1002/advs.202303406

**Published:** 2023-08-07

**Authors:** Xiulei Qi, Yide Liu, Lei Yu, Zhenchuan Yu, Long Chen, Xiankai Li, Yanzhi Xia

**Affiliations:** ^1^ State Key Laboratory of Bio‐Fibers and Eco‐Textiles Collaborative Innovation Center for Marine Biomass Fibers Materials and Textiles of Shandong Province College of Materials Science and Engineering Institute of Marine Biobased Materials Qingdao University Ningxia Road 308 Qingdao 266071 P. R. China

**Keywords:** alginate fibers, bimetallic ions chelation strategy, enhanced flame retardancy, liquid metals, smart textiles

## Abstract

Liquid metal (LM) shows the superiority in smart wearable devices due to its biocompatibility and electromagnetic interference (EMI) shielding. However, LM based fibers that can achieve multifunctional integrated applications with biodegradability remain a daunting challenge. Herein, versatile LM based fibers are fabricated first by sonication in alginate solution to obtain LM micro/nano droplets and then wet‐spinning into LM/alginate composite fibers. By mixing with high‐concentration alginate solution (4–6 wt.%), the LM micro/nano droplets stability (colloidal stability for > 30 d and chemical stability for > 45 d) are not only improved, but also facilitate its spinning into fibers through bimetallic ions (e.g., Ga^3+^ and Ca^2+^) chelation strategy. These resultant fibers can be woven into smart textiles with excellent flexibility, air permeability, water/salt resistance, and high temperature tolerance (−196–150 °C). In addition, inhibition of smoldering result from the LM droplets and bimetallic ions is achieved to enhance flame retardancy. Furthermore, these fibers combine the exceptional properties of LM droplets (e.g., photo‐thermal effect and EMI shielding) and alginate fibers (e.g., biocompatibility and biodegradability), applicable in wearable heating devices, wireless communication, and triboelectric nanogenerator, making it a promising candidate for flexible smart textiles.

## Graphical abstract

Versatile liquid metal/alginate composite fibers are designed by wet‐spinning method through bimetallic ions chelation strategy. With liquid metal droplets in alginate fibers, smoldering is suppressed, which improves the flame retardancy. These fibers with superior flexibility, high/low temperature tolerance, and water/salt resistance, can be woven into smart wearable textiles with photo‐thermal effect, electromagnetic interference shielding, and triboelectric performance.

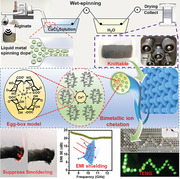

## 1 Introduction

The rapid growth of flexible electronics has propelled the need for smart fibers, as their woven textiles are promising platforms for wearable heating devices, electromagnetic interference shielding, and human‐machine interactive system.^[^ ^1^ ^]^ Wearable electronics featuring good air permeability, biocompatibility, multi‐functionality, and low cost have attracted growing interest. Despite rigid metals (e.g., Au, Ag, and copper)^[^ ^2^ ^]^ with excellent electrical conductivity have been knitted into fabrics for wearable electronics, the mechanical mismatch with human tissues and complicated manufacturing technology remain a daunting challenge. Nonmetallic materials (e.g., graphene,^[^ ^3^ ^]^ carbon nanotubes,^[^ ^4^ ^]^ and MXene)^[^ ^5^ ^]^ are used to circumvent these problems with flexibility and conductivity. However, the ability under stretching/compressing and high cost hamper their scalable applications.

Liquid metals (LMs, e.g., EGaIn with a Ga/In ratio of 75/25 wt./wt., melting temperature of 15.8 °C) with metallic conductivity, favorable flowability, and biocompatibility, show superiority in producing smart wearable textiles.^[^ ^6^ ^]^ LMs have been used in fabricating LM‐based fibers by 3D printing,^[^ ^7^ ^]^ injecting LMs or LM composites into elastic hollow tubes,^[^ ^8^ ^]^ interfacial electrochemical polymerization,^[^ ^9^ ^]^ or coating LMs onto fibers.^[^ ^10^ ^]^ LM fibers possess unique properties, such as excellent electrical conductivity, intrinsic stretchability, chemical activity, and self‐healing ability, providing great potential for wearable devices, soft sensors, variable stiffness electrodes, and shape memory fibers compared with conventional fibers.^[^ ^6b^ ^]^ However, these fibers generally suffer from conductivity degradation during utilization, thereby decreasing the functionality. In addition, ultrahigh surface tension (EGaIn: 624 mN m^−1^) of LMs complicates the handing and processing, resulting in the leakage during the fabrication of fibers. Nanometerization of LMs by sonication to obtain LMs micro/nano droplets could decrease the surface tension and be applied to fabricate LM‐based fibers. For instance, LM microfibers with sheath‐core structure were fabricated by triaxial wet‐spinning method and recovered conductivity by “freezing‐plus‐stretching” strategy to sinter LM particles.^[^ ^11^ ^]^ MXene‐reinforced LM‐based polymer fibers via interface engineering were also constructed by a simple, economical and controllable extrusion method, showing great potential for wearable multifunctional textiles.^[^ ^12^ ^]^ Moreover, the advantages and innovative aspects of LM fibers’ excellent transformability and knittability make it possible to develop more intelligent fibers and devices with the characteristics of self‐heating, self‐powering, wireless charging, mechanical sensing, and switchable conductivity.^[^ ^6b^ ^]^ Nevertheless, there is great challenge in balancing biodegradability, biocompatibility, durability, and conductivity for constructing LM‐based fibers.

Alginate fibers featuring softness, biocompatibility, and biodegradability, are ideal material for manufacturing wearable electronics, which are formed by wet‐spinning method through the chelation of alginate with divalent cations (e.g., Ca^2+^ and Mg^2+^).^[^ ^13^ ^]^ Alginate is a natural unbranched binary copolymer of *β*‐(1‐4)‐linked–_D_‐mannuronic acid (M) and *α*–_L_‐guluronic acid (G) groups extracted from brown algae, which is highly soluble in water. Moreover, alginate, as a dispersion reagent, could stable LM droplets by forming microgel shells via coordination of carboxyl groups with Ga ions, thereby affording biocompatible aqueous LM inks for flexible electronics.^[^ ^14^ ^]^ Furthermore, LM/calcium alginate hydrogels were introduced by developing LM droplets into biocompatible calcium alginate cross‐linked network, showing pretty flexible and controllable functions in endovascular embolization and tumor embolotherapy.^[^ ^15^ ^]^ These endeavors confirmed the possibility that LM fibers were constructed by dispersing LM into alginate aqueous solution and fabricating fibers by the subsequent wet‐spinning method.

Previous study showed that EGaIn nanodroplets wrapped with microgel shells formed by the coordination of carboxyl groups of alginate and Ga^3+^ could maintain colloidal stability in air for only 7 days.^[^ ^14^ ^]^ However, owing to the ultrasonic cavitation effect result in the broken of molecular entanglement of alginate, the viscosity decreased quickly,^[^ ^16^ ^]^ which can't meet the requirement of spinning. In this study, mixing LM dispersions with high concentration (4–6 wt.%) of alginate solution will not only improve the colloidal stability (> 30 d) in air resulted from the balance of buoyancy and gravity, but also facilitate its spinning as wet‐spinning dope. Subsequently, versatile LM/alginate fiber was constructed by a conventional wet spinning method through the formation of chelating “egg‐box” networks.^[^ ^17^ ^]^ During wet‐spinning process, CaCl_2_ solution was used as coagulation bath, providing Ca^2+^ as cross‐linking agent to cheat with carboxyl groups of alginate. Thus, alginate fiber was produced by bimetallic ion cross‐linking strategy, in which alginate was cross‐linked by internal Ga^3+^ coated on LM droplets and external Ca^2+^, providing excellent flame retardancy with limit oxygen index of 40.

In addition to stabilizing LM droplets in aqueous solution, alginate assisted these LM droplets in spinning into versatile LM fibers. These LM/alginate composite fibers exhibited superior biocompatibility, biodegradability, and air permeability, offering potential of being woven into textiles with integrated functions of electromagnetic interference shielding, photo‐thermal effect, and energy generation. In addition, these composite fibers could undergo various deformations and harsh environments (e.g., low/high temperature), making them suitable for smart textiles. Thus, the combination of scalable wet‐spinning technology and excellent performance of LM fibers not only broaden the application of LM and alginate fiber, but also paves the way to multi‐functional wearable electronics and smart textiles.

## 2 Results and Discussion

### 2.1 Design of Ultra‐High Colloidal and Chemical Stable LM Droplets

**Figure 1:**
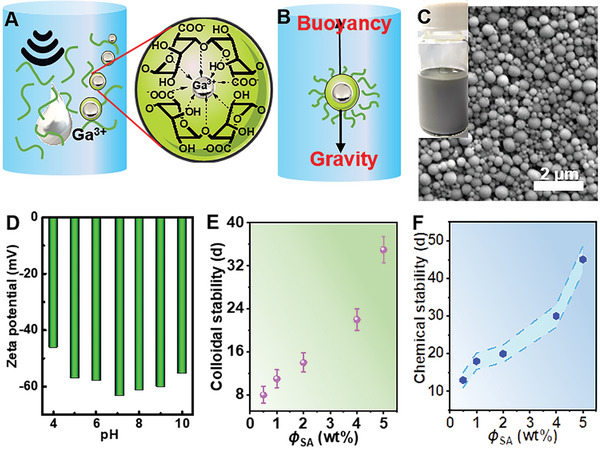
Design of high stability of LM dispersions. Schematic description of sonicating EGaIn in A) alginate solutions and shelled in alginate microgel and B) the balance analysis in dispersions for LM droplet. C) SEM image of LM droplets and its aqueous dispersion in the inset. D) Zeta potential of LM droplets shelled in alginate microgel at different pH values. LM dispersions was diluted to 0.5 mg mL^‒1^ for measurements. E) Colloidal stability and F) chemical stability of LM/SA dispersions at different alginate concentrations (1.0–5.0 wt.%) after mixing. LM concentration: 20 mg mL^‒1^.

Typically, LM droplets are not stable under aqueous solution owing to the large density (EGaIn, 6.28 g mL^‒1^) and chemical activity toward oxygen and water. Though cheating with carboxyl groups of alginate (**Figure** **1**A), EGaIn nanodroplets could not maintain stability >7 days in air.^[^ ^14^ ^]^ The balance between gravity and buoyancy is important for improving colloidal stability (Figure 1B), which is related to the viscosity of alginate and density of LM droplets.

During the sonication process in alginate solution, LM was ruptured into small droplets by the shear forces result from rapidly collapsing bubbles (Figure 1C; Figure S1, Supporting Information). Meanwhile, collisions between polymers molecules and solvent molecules would accelerate the broken of C─C bond and the interruption of macromolecular segment and entanglement, resulting in the decreased viscosity (Figure 1C; Figure S1B, Supporting Information) and reduced molecular weight.^[^ ^16a^ ^]^ When considering colloidal stability, the force of droplet in colloidal solution is identified as:

(1) $$F=3\pi \eta dv_{0}-\frac{1}{6}\pi d^{3}\left(\rho -\rho_{0}\right)g$$

where the first and second terms are the force arising from resistance and sedimentation force, respectively. *η* is the kinetic viscosity of the suspension, *v*_0_ is the sedimentation rate of the LM droplet, *ρ*_0_ is the density of SA solution, *d* is the diameter of LM droplets, *ρ* is the density of LM droplets, and *g* is the acceleration of gravity. When LM droplets are stable, F = 0, the sedimentation rate could be represented as:

(2) $$v_{0}=\frac{d^{2}\left(\rho -\rho_{0}\right)}{18\eta }g$$

Therefore, colloidal stability could be enhanced by decreasing LM droplets sizes and increasing the viscosity of suspension (for detail in Supporting Information). However, previous study showed that much longer sonication time and high concentration of alginate solution could not reduce droplet size as low as 125 nm.^[^ ^14^ ^]^ Thus, for LM in alginate system, sonication at low concentration (0.5 wt.%) and the following mixing with high concentration (6 wt.%) were adopted to obtain smaller LM droplets (average diameter of ≈150 nm, Figure 1C; Figure S2, Supporting Information) and high viscosity (18.5 Pa s) of suspension in order to achieve higher colloidal stability.

Similarly, LM droplets were encapsulated into micogel by the coordination of carboxylic groups within alginate G segments and Ga^3+^ produced from oxidizing Ga. In addition, the suspension at high concentration (4.6 wt.%) alginate solution after mixing exhibited excellent colloidal dispersion stability with zeta potential >42 mV even under acidic or alkaline (4 < pH < 10) conditions (Figure 1D). The robust gel shell within 10 nm in high concentration (4.6 wt.%) alginate solution not only improved the colloidal stability (> 30 d, Figure 1E; Figures S3 and S4, Supporting Information), but also retarded the further oxidation, thereby offering high chemical stability (> 45 d, Figure 1F). This high stability offers the potential in storing, enabling further applications in wet‐spinning.

### 2.2 Wet‐Spinning of LM/Alginate Composite Fibers

**Figure 2:**
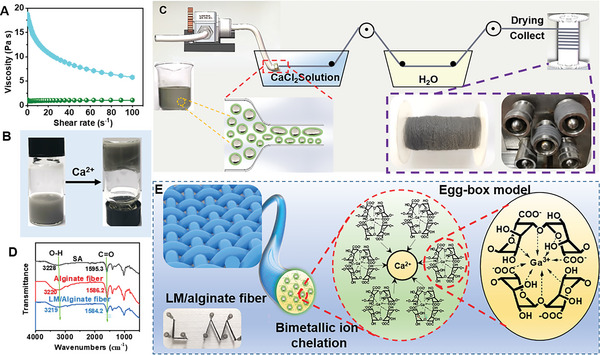
Wet‐spinning of LM/alginate composite fibers. A) Steady rheological properties of LM/SA dispersion obtained by ultrasonication (*ϕ*_SA_ = 4.6 wt.%) and ultrasounication at *ϕ*_SA_ = 0.5 wt.% with mixing at *ϕ*_SA_ = 6 wt.%. LM: 20 mg mL^‒1^. B) Gelation of LM/SA dispersion by Ca^2+^. C) Schematic illustration of the wet‐spinning process of the LM/alginate composite fiber. D) FTIR spectra of SA, alginate fiber, and LM/alginate composite fiber. E) Schematic mechanism for LM/alginate composite fibers enabled by egg‐box model and bimetallic ion chelation strategy.

As is observed in non‐Newtonian fluid characterized by shear thinning, the viscosity of suspensions prepared by sonication and blending method decreased with an increase in the shear rate (**Figure** **2**A), in contrast with constant viscosity for low concentration alginate sodium (SA) solution. The viscosity (18.5 Pa s) of suspensions meets the requirement of spinning solution,^[^ ^17^ ^]^ enabling these solution be extruded through spinneret holes to form filaments. Ca^2+^ was used as cross‐linking agent in wet‐spinning for manufacturing LM/alginate fibers. The gelation ability of Ca^2+^ was confirmed by the vial inversion method (Figure 2B). As shown in Figure 2C, the as‐prepared LM/SA dispersions as spinning dope was extruded into a coagulation solution containing Ca^2+^ and then washed in water to obtain continuous fibers by a reel. FTIR was used to verified the chelation of metal ions and alginate (Figure 2D). The peak at 3328 cm^‒1^ revealed the stretching vibrations pf O─H, and the peak at 1596 cm^‒1^ originated from stretching vibrations of carboxyl group (COO^‒^) shifted to 1584 cm^‒1^, when bimetallic ions (i.e., Ga^3+^ and Ca^2+^) cheated with carboxyl groups.^[^ ^13^, ^18^ ^]^ The coordination between bimetallic ions and carboxyl group holds the alginate molecules together, resulting in a firm gel fibers with two‐deck “egg‐box” structure (Figure 2E).

**Figure 3:**
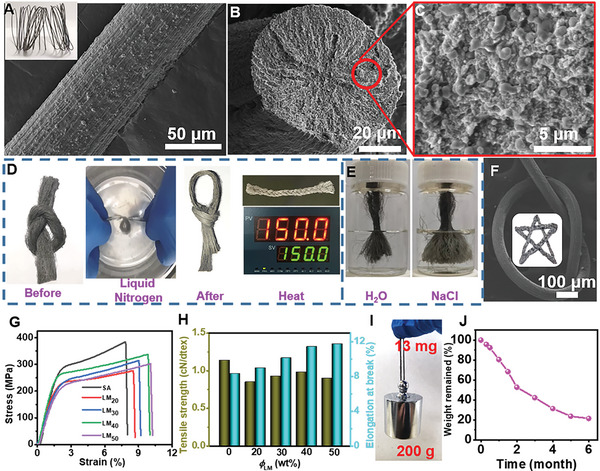
Physical characterization and mechanical property of LM/alginate composite fibers. SEM image of A,B) composite fiber and C) enlarged cross‐section of LM droplets. D) High/Low temperature tolerance and E) water/salt resistance of LM/alginate composite fibers. F) SEM image of two knotted fibers and braided pattern. G) Stress–strain curves and H) mechanical property of composite fibers with different content of LM (e.g., LM_40_ represents the LM content of 40 wt.%). I) Visual observation of fibers upon lighting weights. J) Time dependence of weight loss for biodegradation of composite fibers in soil. The inset gives its visual observation after six months degradation.

LM/alginate fibers were wet‐spun with a cross‐section of circular (diameter of 70 µm) as shown in scanning electron microscopy (SEM) (**Figure** **3**A,B). And these fibers with tuned diameter of 20–120 µm controlled by the diameter of spinneret orifice could be large‐scale preparation (Figure S5, Supporting Information). LM droplets are evenly distributed on the surface which are demonstrated by the corresponding element mapping (Ga and Ca) image (Figure S6, Supporting Information). Meanwhile, owing to the stable dispersions during spinning, these LM droplets with content of 10–50 wt.% were also embedded inside the fibers uniformly (Figure 3C; Figure S7, Supporting Information). LM/alginate fibers would retain flexibility and morphological integrity (Figure 3D; Figure S8, Supporting Information) even when exposed to low temperature (i.e., liquid nitrogen at −196 °C, Video S1, Supporting Information) or high temperature (e.g., 150 °C), which could be attributed to the LM droplets’ superior thermal conductivity.^[^ ^19^ ^]^ In addition, there were no apparent changes of color or decreased integrity under washing in water and salt solution (Figure 3E; Figure S9, Supporting Information), showing high stability of LM droplets and promising potential in practical applications. These resultant fibers with excellent flexibility could be knotted and woven into various interesting shapes and fabrics (Figure 3F).

In terms of tensile strength and stress at break, an optimized value of strength of 1.4 cN dtex^‒1^ and elongation at break of 11.5% were achieved at the LM content of 40 wt.% in Figure 3G,H, which was comparable to alginate fibers. Owing to the presence of LM droplets with superior fluidity and flexibility, the elongation at break was improved, which can be ascribed to energy dissipation result from the deformability of soft LM droplets during stretching. Thanks to the super mechanical properties of LM/alginate composite fibers, these fiber could undergo a weight which was 15 000 times heavier than itself (Figure 3I). In addition, these resultant fibers could be woven into fabrics with high air permeability (Figure S10, Supporting Information), hydrophilic surfaces (Figure S11, Supporting Information), and adsorption properties for ionic dyes (e.g., absorption efficiency of 87% for methylene blue, Figure S12, Supporting Information), demonstrating its promising potential in smart textiles. Furthermore, these fibers could be degraded ≈80% buried in the soil for six months (Figure 3J), showing unprecedented ability of biodegrading.

### 2.3 Flame Retardancy of LM/Alginate Composite Fibers

Alginate fibers featuring better flame retardancy, have been applied in food industry, textile industry, and biomedical textile.^[^ ^20^ ^]^ However, smoldering with serious damage of alginate fibers may limit its application in fire retardant protective textiles.^[^ ^21^ ^]^ Interestingly, by introducing LM droplets into alginate fibers, it was found the smoldering problem was suppressed significantly.

**Figure 4:**
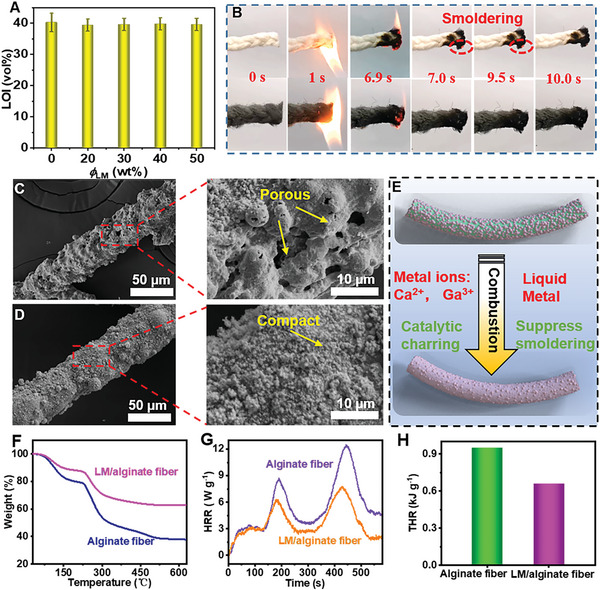
Flame retardancy and combustion behaviors of composite fibers. A) LOI values and B) vertical combustion test of alginate fibers and LM/alginate composite fibers. SEM images of the carbon residue after the micro‐calorimetry test for C) alginate fibers and D) LM/alginate composite fibers. E) Flame retardant mechanism of LM/alginate composite fibers. F) TGA, G) HRR, and H) THR curves of alginate fibers and LM/alginate fibers.

First, limit oxygen index (LOI) results (> 40) showed almost the same flame retardancy after introducing LM droplets (**Figure** **4**A). During the burning test for alginate fibers, in spite of having no afterglow (flameless combustion) after leaving the fire, there will be a noticeable afterflame (flame combustion) and smoldering with duration time >3 s (Figure 4B and Video S2, Supporting Information). This may be caused by porous carbon during combustion created by a “narrow tube effect”, in which heat tends to be concentrated rather than diffused outward. Besides this, smoldering occur until the temperature down to the temperature of thermal decomposition, which brought great danger in fire safety (Figure 4C). In contrast, LM/alginate fibers were extinguished immediately without smoldering phenomenon after leaving the flame (Figure 4B). On the one hand, LM droplets inside of fibers remain spherical state owing to the robust shells, which maintain the integrity of fibers. LM on the surface of these fibers would melt under high temperature, coating on the surface of carbon layer uniformly (Figure 4D; Figure S13, Supporting Information), preventing the flame from burning inside and the diffusion of heat. On the other hand, the presence of bimetallic ions (Ga^3+^ and Ca^2+^) are also conducive to flame retardancy and inhibition of smoldering (Figure 4E).^[^ ^22^ ^]^

To further evaluate fire resistance of these fibers, thermogravimetric (TG) analysis, and micro‐calorimetry based on the oxygen consumption to determine the combustion performance are introduced. Owing to the high boiling point of LM droplets (2000 °C),^[^ ^23^ ^]^ the residue was almost LM in TG curve (Figure 4F). The heat release rate (HRR) and total heat release (THR) of LM/alginate composite fibers are obviously better than those of alginate fibers, with a lower peak HRR of only 59.9% for alginate fibers. And THR is also reduced 30.5% compared with alginate fibers (Figure 4G,H). These results indicated that LM/alginate composite fibers exhibited excellent flame retardant performance with the suppression of smoldering, making these resultant fibers suitable for a wider application scenarios.

### 2.4 Photo‐Thermal Properties, EMI Shielding Performance, and Triboelectric Performance of Fibers

**Figure 5:**
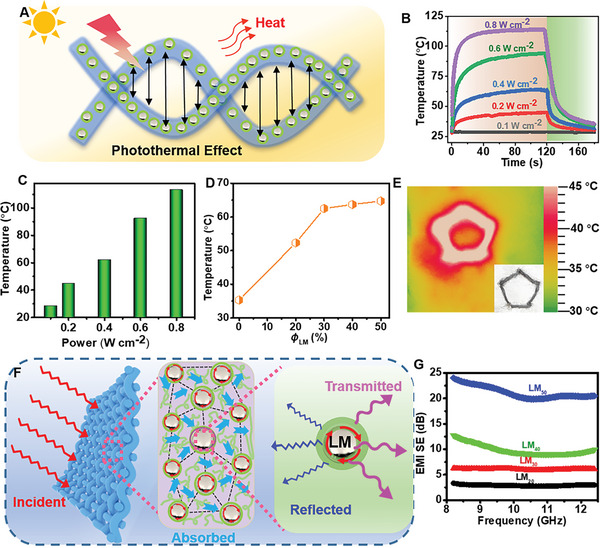
Photo‐thermal and EMI shielding performance of resultant fibers. A) Schematic illustration of photo‐thermal effect of LM droplets in composite fibers. B) Temperature–time curve and C) maximum temperature of LM/alginate composite fiber (*ϕ*_LM_ = 40 wt.%) under NIR irradiation with power densities. D) Maximum temperature for LM/alginate composite fibers with different LM contents. Power density of NIR: 0.4 W cm^‒2^. E) Optical image (inset) and its infrared temperature distribution image at 120 s of composite fibers. Power density of NIR: 0.2 W cm^‒2^, *ϕ*_LM_ = 40 wt.%. F) EMI shielding mechanism of LM droplets in composites fibers. G) EMI SE_T_ of LM/alginate fibers with various LM contents. (e.g., LM_40_ represents the LM content of 40 wt.%).

LM nanodroplets with superior photo‐thermal effect are reported in photo‐thermal therapy field owing to its outstanding plasmonic effect (**Figure** **5**A).^[^ ^24^ ^]^ Similarly, there might exist LM droplets encapsulated inside the composite fiber with a diameter of < 100 nm, whose photo‐thermal effect had been further studied. When exposed to a near‐infrared light radiation (wavelength of 808 nm), the temperature was raised at different powers (Figure 5B), proving the strong photo‐thermal effect of LM droplets. For instance, LM/alginate composite fibers (ϕ_LM_ = 40 wt.%) could be heating up to 120 °C within 50 s at power density of 0.8 W cm^‒2^, in sharp contrast with alginate fibers (the elevated temperature of 20 °C) (Figure 5C). It was also found in Figure 5D that photo‐thermal effect was related to the content of LM. As increase LM content in composite fibers, the increased temperature and heating rate rises, demonstrating the high photo‐thermal conversion ability of LM droplets. Moreover, these result fibers showed a stable photo‐thermal effect even under hundreds of utilization or after eight months of storage (Figure S14, Supporting Information). Besides these, when woven into fabric, these fibers are capable of warming people when exposed to sunlight (Figure 5E), which broadened the applications of thermal management devices.

With the presence of LM droplets in fibers, these composite fibers also exhibited an EMI shielding property with a shielding efficiency (SE) of 24 dB in the X‐band frequency range of 8.2–12.4 GHz (Figure 5F,G), which meet the requirement of commercial shielding materials (20 dB).^[^ ^25^ ^]^ Compared with alginate fibers with SE of only 1.57 dB (Figure S15, Supporting Information), the superior EMI shielding performance of LM/alginate composite fibers are attributed to the absorption of alginate molecular chains and reflection of LM droplets embedded in the core‐shell structure.^[^ ^26^ ^]^ Thus this composite fibers would offer an alternative for smart textiles with EMI shielding property.

**Figure 6:**
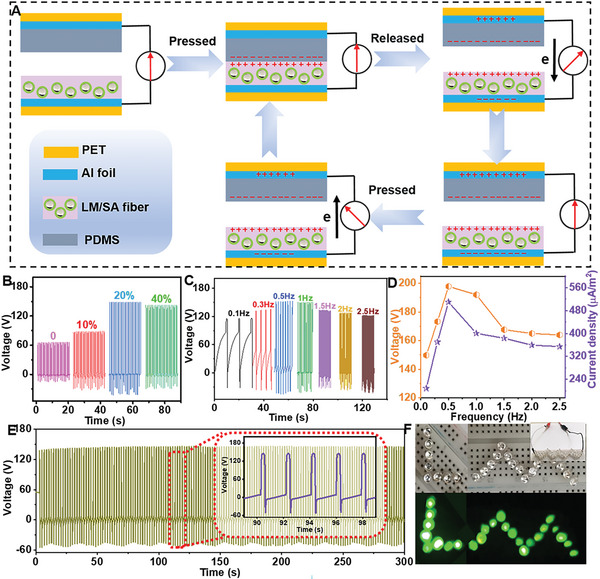
Application of LM/alginate fibers/fabrics in TENG. A) Working mechanism of LM/alginate composite fibers based TENG. B) Output voltage of LM/alginate fibers based TENG with different contents of LM at the motion frequency of 0.5 Hz. C) Output voltage and D) current density of LM/alginate fibers based TENG at different frequencies. (*ϕ*_LM_ = 20 wt.%). E) Stability of TENG performance at frequency of 0.5 Hz. (*ϕ*_LM_ = 20 wt.%). F) Pattern LED bulbs were lightened by TENG.

LM/alginate composite fibers with electropositive surface relative to polydimethylsiloxane (PDMS) could act as high performance triboelectric nanogenerators (TENG).^[^ ^27^ ^]^ As shown in **Figure** **6**A, composite fibers and PDMS were used as two tribo‐material layers in TENG test configuration. When they are forced in contact, the opposite static charges with equal electric density will be accumulated on the inner surface because of contact electrification. When they are separated, the electrons will move from Al foil on PDMS side to Al foil on another side of the fiber fabric, driving under the effect of electrostatic induction. Thus, a positive current will be generated upon separating and a negative electrical signal will occur when they are pressed, in which these iterative processes can produce alternating current.

The induced voltage and current with LM/alginate composite fibers and alginate fibers by repeatedly contacting were monitored. In comparison to output voltage of 90 V for alginate fiber, composite fibers could be achieved the maximum output voltage of 198 V (Figure 6B,C), showing the enhancement in TENG performance of LM droplets caused by enhanced charge trapping properties result from the increase interface state density generated at the surface of fibers. (Figure S16A, Supporting Information).^[^ ^27c^ ^]^ However, much more LM contents (> 20 wt.%) can't increase TENG performance continuously, which may be because electrons hop from one site to another resulting in small gap between LM droplets on the surface of fibers. (Figure S16B, Supporting Information). In addition, the output voltage and current density could be optimized as 198 V and 510 µA m^‒2^ at a frequency of 0.5 Hz, respectively (Figure 6D). TENG performance will be improved as the increase of frequency (< 0.5 Hz), which may be caused by the increment of effective contact area. But, high frequency (> 0.5 Hz) will reduce output voltage and current result from the possible smaller deformation time.^[^ ^28^ ^]^ The LM/alginate fibers‐based TENG with superior productivity stability could light dozens of LED bulbs with the pattern of “LM” (Figure 6E,F), demonstrating the power supply ability, and it is applicable in mobile devices, wired and wireless power transmission systems. When utilized as smart fabrics, these fibers could remain excellent performance even encountering washing, rubbing and bending circumstances, and could not detach at stress concentration points (e.g., knots, Figure S17, Supporting Information), which exhibits its promising potential in wearable textiles.

## 3 Conclusion

In summary, we showed that sonicating LM in alginate solution and mixing LM micro/nano droplets with high concentration alginate solution (4–6 wt.%) could improve the colloidal and chemical stability more than one month, which enabled LM dispersions as spinning dope. Through this facile wet‐spinning process, LM/alginate composite fibers were fabricated by bimetallic ions (e.g., Ga^3+^ and Ca^2+^) chelation strategy. Due to the presence of LM droplets and bimetallic ions, LM/alginate composite fibers exhibited super flame retardancy performance with LOI >40 and no smoldering problem. With LM droplets dispersed uniformly in fibers, these resultant fibers showed unique property, such as photo‐thermal effect and EMI shielding. In addition, these versatile fibers could be easily woven into multifunctional textiles, which presented superior flexibility, high air permeability, high/low temperature tolerance, and water washability. Benefiting from charge trapping properties of LM droplets, we further demonstrate the applications of LM/alginate fibers in TENG, which may broaden the application of LM and alginate fibers in smart wearable textiles. In addition, biodegradable LM/alginate fibers could achieve excellent composite properties, which meet the concept of environmental protection and sustainable development.

## 4 Experimental Section

### Materials

EGaIn (75.5 wt.% Ga and 24.5 wt.% In with a melting point of 15.8 °C) was purchased from Shenyang Jiabei Trading Co., Ltd. (China). Sodium alginate (weight‐average molecular weight, Mw ≈ 200 000−300 000) was purchased from Aladdin Industrial Co. Ltd. (China). Anhydrous calcium chloride (analytical pure AR) was purchased from Sinopharm Chemical Reagent Co., Ltd. (China). All solutions were prepared with ultrapure water (Resistivity: 18.2 MΩ cm^‒1^)

### Preparation of LM Dispersions as Spinning Dope

Typically, bulk EGaIn (280 mg) was dispersed into aqueous solution (7 mL) of 0.5 wt.% sodium alginate by sonicating the mixture with a probe sonicator (JY92‐IIN; Power 900 W with 80% amplitude) in ice water bath for 20 min. Sodium alginate aqueous solution (6 wt.%, 21 mL) was added into the as‐prepared dilute LM dispersions and stirred (500 r min^‒1^) for 5 min to obtain LM/SA homogeneous solutions. The dosage of EGaIn was tuned to adjust the mass fractions of LM.

### Wet‐Spinning of LM/Alginate Composite Fibers

The as‐prepared LM/SA homogenous dispersions as spinning dope were defoamed in a spinning tank and extruded quantitatively by a metering pump (500 mL h^‒1^) with a pressure of 200 kPa and then passed through a spinneret into coagulation bath (4 wt.% CaCl_2_ solution). Then the fibers were stretched at a velocity of 3 m min^‒1^, washed in water, and collected on a roller. These fibers were dried in the air and could be further knitted into fabrics.

### Measurement of EMI Shielding

The EMI shielding performances of fabric knitted by alginate fibers and LM/alginate composite fibers were tested by a vector network analyzer (Keysight E5063A, China) in the frequency range of 8.2–12.4 GHz.

### Fabrication of TENG

Polydimethylsiloxane (PDMS) and fiber fabric with the same size (1 cm × 4 cm × 0.5 mm) separated by glass sheets (1 cm × 2 mm × 1 mm) were adhered to aluminum foil as positive and negative friction layer on the TENG electrode, respectively. Both layers were further assembled on the PET support layers. The dynamic fatigue tester with a robotic arm (LinMot) that could control impact frequency, distance, and contact force was utilized in TENG test. And the output voltage and current were recorded with a digital source meter (KEITHLEY, China).

### Characterization

The colloidal stability of LM micro/nano droplets was characterized by a nanoparticle size‐zeta potentiometer (Nano ZSE, Malvern, United Kingdom). The crystal structure of the samples was characterized by X‐ray diffractometer (XRD, Smart Lab 3KW) using Cu Kα radiation. Morphology, microstructure, and elemental distribution were analyzed with a field emission scanning electron microscope (FESEM, Quanta250 FEG, FEI) and transmission electron microscope (TEM, H‐7650, Hitachi). Static rheological characterization of SA and LM/SA colloidal solutions was measured using a dynamic viscoelastic spectrometer (DHR3, TA, United States). The FT‐IR spectroscopy was tested using a FTIR spectrometer (Nicolet 5700, Shanghai, China). High temperature resistance of fibers using far‐infrared microcrystalline electric heating plate (DB‐XWJ, Shanghai, China). Mechanical properties of the samples were tested using a universal testing machine (WDW‐2T, Jinan, China) and automatic single fiber tester (Favimat‐airobot, TEXTECHNO, Germany) at a strain rate of 20 mm min^−1^. Contact angles were recorded using a Theta (Biolin, Sweden) goniometer. Evaluation of the adsorption performance of fibers to MB solution was by UV/vis spectrophotometer (T9, Beijing, China). The limited oxygen index (LOI) was measured by a HC‐2C oxygen index meter (Nanjing, China). Thermogravimetric analysis (TGA) was carried out to determine the content of components by a thermal gravimetric analyzer (Mettler Toledo TGA‐2) in Ar atmosphere with a heating rate of 10 °C min^‒1^ from 30 to 800 °C. Micro calorimeter (PCFC, FTT, United Kingdom) was used to test the combustion properties of fibers.

## Conflict of Interest

The authors declare no conflict of interest.

## Supporting information

Supporting Information

Supplemental Video 1

Supplemental Video 2

## Data Availability

The data that support the findings of this study are available from the corresponding author upon reasonable request.
