# Supplementary material for: Versatile Liquid Metal/Alginate Composite Fibers with Enhanced Flame Retardancy and Triboelectric Performance for Smart Wearable Textiles
Source: Adv Sci (Weinh). 2023 Aug 7;10(29):2303406. doi: 10.1002/advs.202303406 (PMC10582420; doi:10.1002/advs.202303406)
Supplement: Supplementary file 1 — Supporting Information [file ADVS-10-2303406-s003.pdf]

ADVANCED SCIENCE
Open Access

Supporting Information

for *Adv. Sci.*, DOI 10.1002/advs.202303406

Versatile Liquid Metal/Alginate Composite Fibers with Enhanced Flame Retardancy and Triboelectric Performance for Smart Wearable Textiles

Xiulei Qi, Yide Liu, Lei Yu, Zhenchuan Yu, Long Chen, Xiankai Li and Yanzhi Xia**

Supporting Information

Versatile liquid metal/alginate composite fibers with enhanced flame retardancy and triboelectric performance for smart wearable textiles

Xiulei Qi, Yide Liu, Lei Yu, Zhenchuan Yu, Long Chen, Xiankai Li and Yanzhi Xia**

Table of contents in Supporting Information:

Stability analysis of LM droplets

Supporting figures

Figure S1–Figure S17

Supporting videos

Video S1 –Video S2

Stability analysis of LM droplets

The sedimentation force F_1 of LM droplets in colloidal solution can be estimated analytically by the following equation:

$$F_1 = \frac{1}{6}\pi d^3(\rho - \rho_0)g \quad (1)$$

where ρ_0 is the density of SA solution, d is the average diameter of LM droplets, ρ is the density of LM droplets, and g is the acceleration of gravity. According to Stokes law, the resistance F_2 of the droplets during sedimentation can be expressed as follows:

$$F_2 = 3\pi\eta d v_0 \quad (2)$$

Where η is the kinetic viscosity of the suspension, v_0 is the sedimentation velocity of the LM droplet. When the LM droplets are stable in the initial state, which is F_1 = F_2, the sedimentation rate can be estimated analytically by the following equation:

$$v_0 = \frac{d^2(\rho - \rho_0)}{18\eta}g \quad (3)$$

If assuming d = 150 nm, ρ = 6.3 g mL^{-1}, g = 9.8 N Kg^{-1}.

For diluted alginate solution (e.g., 0.5 wt%), η = 0.08 Pa s, ρ_0 = 1.0 g mL^{-1}, LM droplets sedimentation velocity is v_1 = 8.1×10^{-10} m s^{-1}.

For spinning dope after mixing with high concentration alginate solution (6.0 wt %), ρ_0 = 1.04 g mL^{-1} , η = 18.5 Pa s, LM droplets sedimentation velocity is v_2 = 3.5×10^{-12} m s^{-1}.

Therefore, after mixing with high concentration alginate solution, the sedmimention velocity of LM droplets is much lower than the diluted alginate solution, which improve the colloidal stability significantly.

Supporting figures

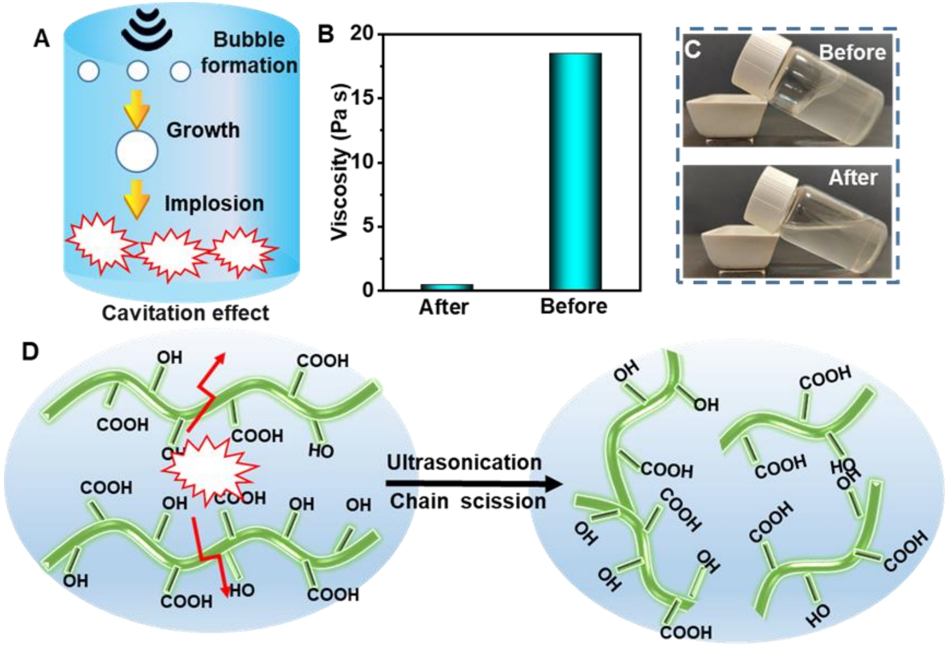

Figure S1. The effect of ultrasonication on alginate solution. (A) Schematic illustration of ultrasonication effect in the solution. (B–C) Comparison of viscosity (B) and visual image (C) of sodium alginate solution (4.6 wt%) before and after ultrasound. (D) Schematic illustration of the ultrasonic cavitation effect result in the broken of molecular entanglement of alginate chains.

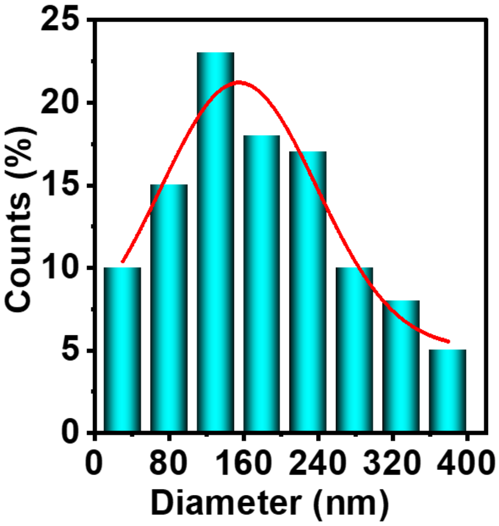

Figure S2. Diameter histogram of LM droplets.

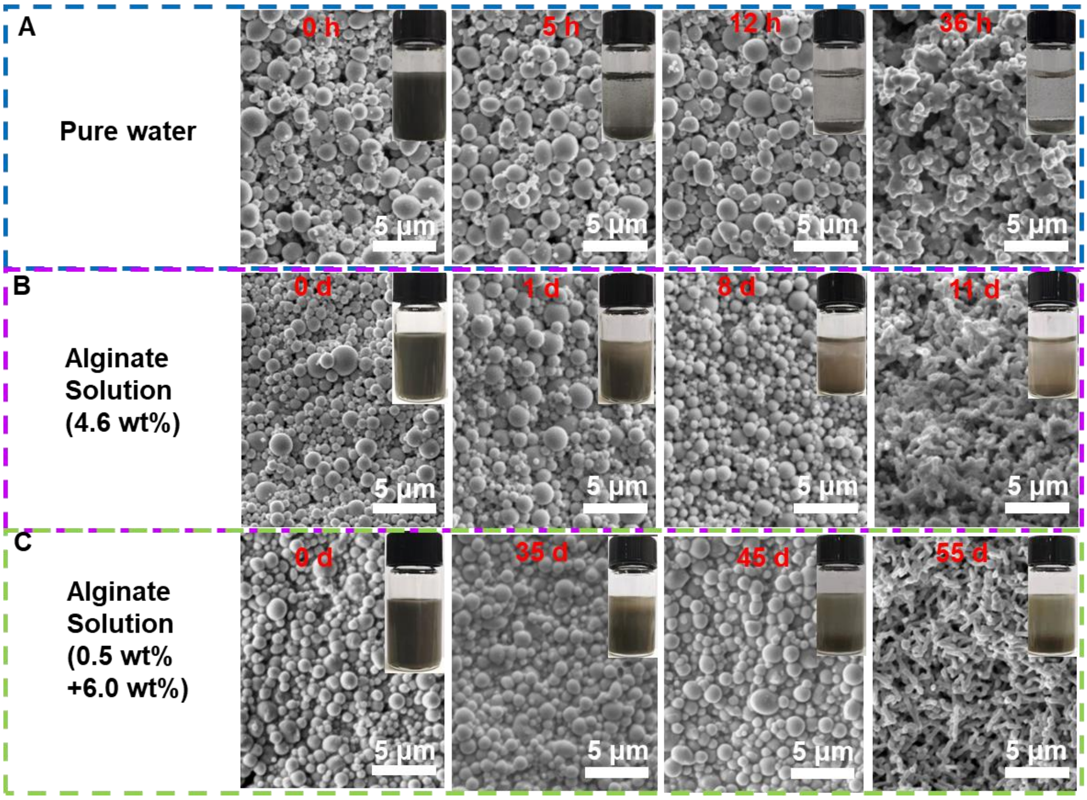

Figure S3. Comparison of the stability of LM in sonication in different solutions. (A & B) SEM image of LM droplets sonicated in pure water (A) and 4.6 wt% alginate solution (B) and stored in different periods. (C) SEM images of LM droplets sonication in 0.5 wt% alginate solution and then mixing with 6.0 wt% alginate solution in different period. The insets are the corresponding photographs of LM dispersions. LM concentration: 20 mg mL^{-1}.

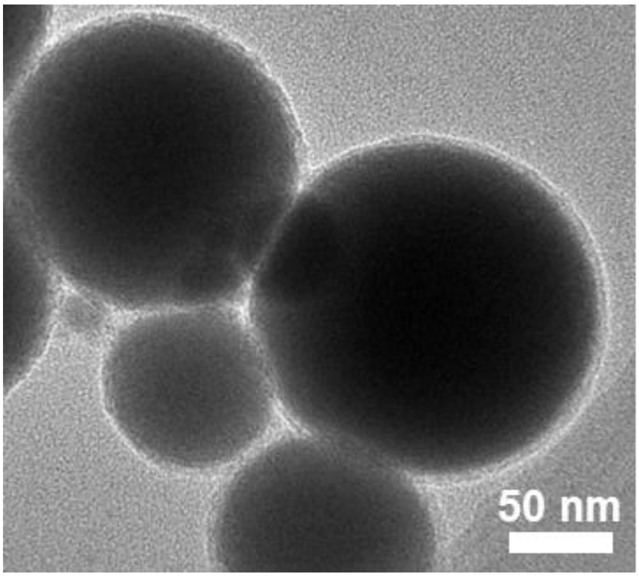

Figure S4. Typical TEM image of LM droplets.

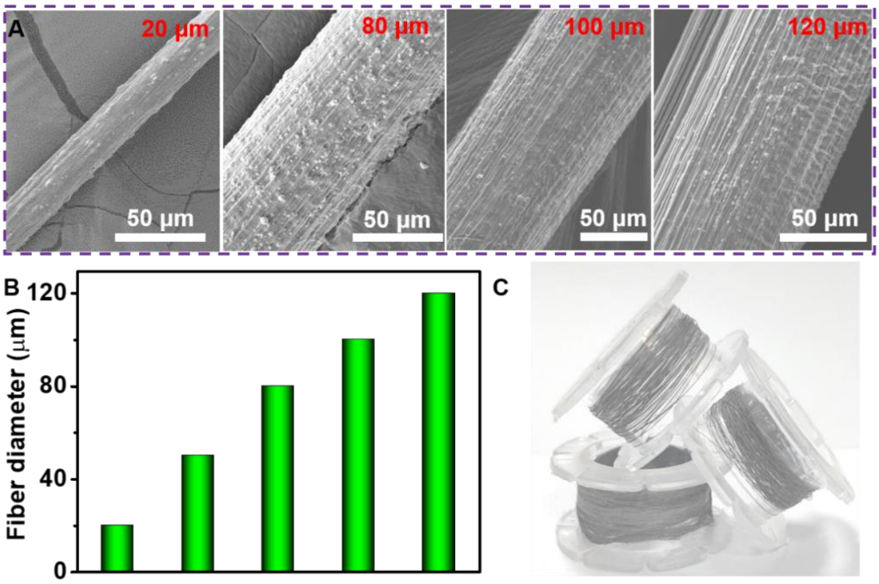

Figure S5. (A) SEM image of LM/alginate composite fibers of different diameters. (B) Diameter distribution of LM/alginate composite fibers. (C) Optical images of LM/alginate fibers in large scale preparation.

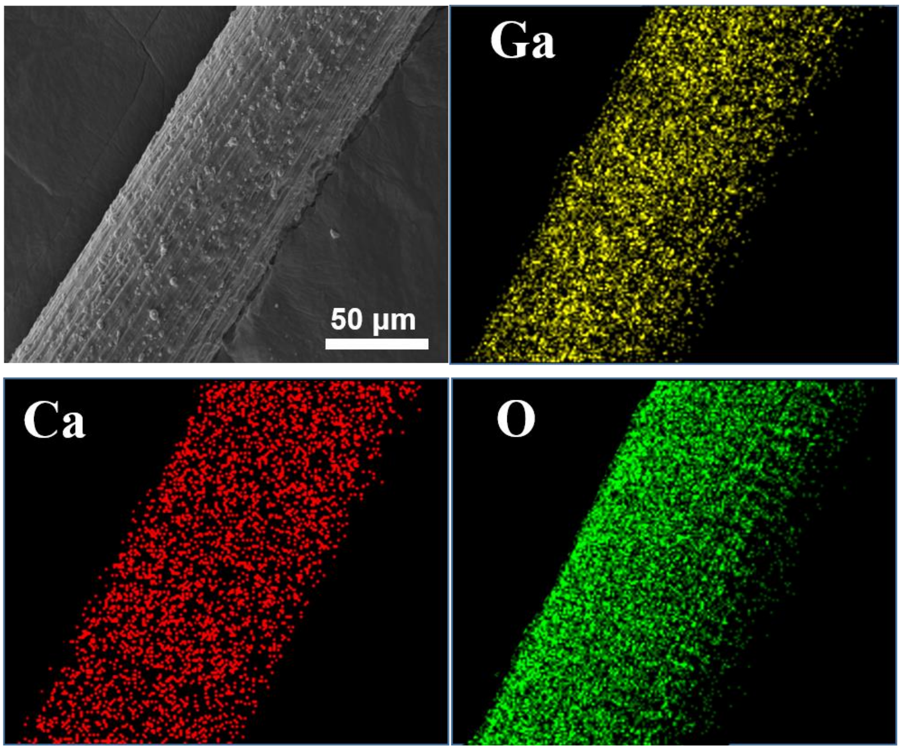

Figure S6. Typical SEM image and element mapping (Ga, Ca and O) of LM/alginate composite fibers. LM content: 40 wt%.

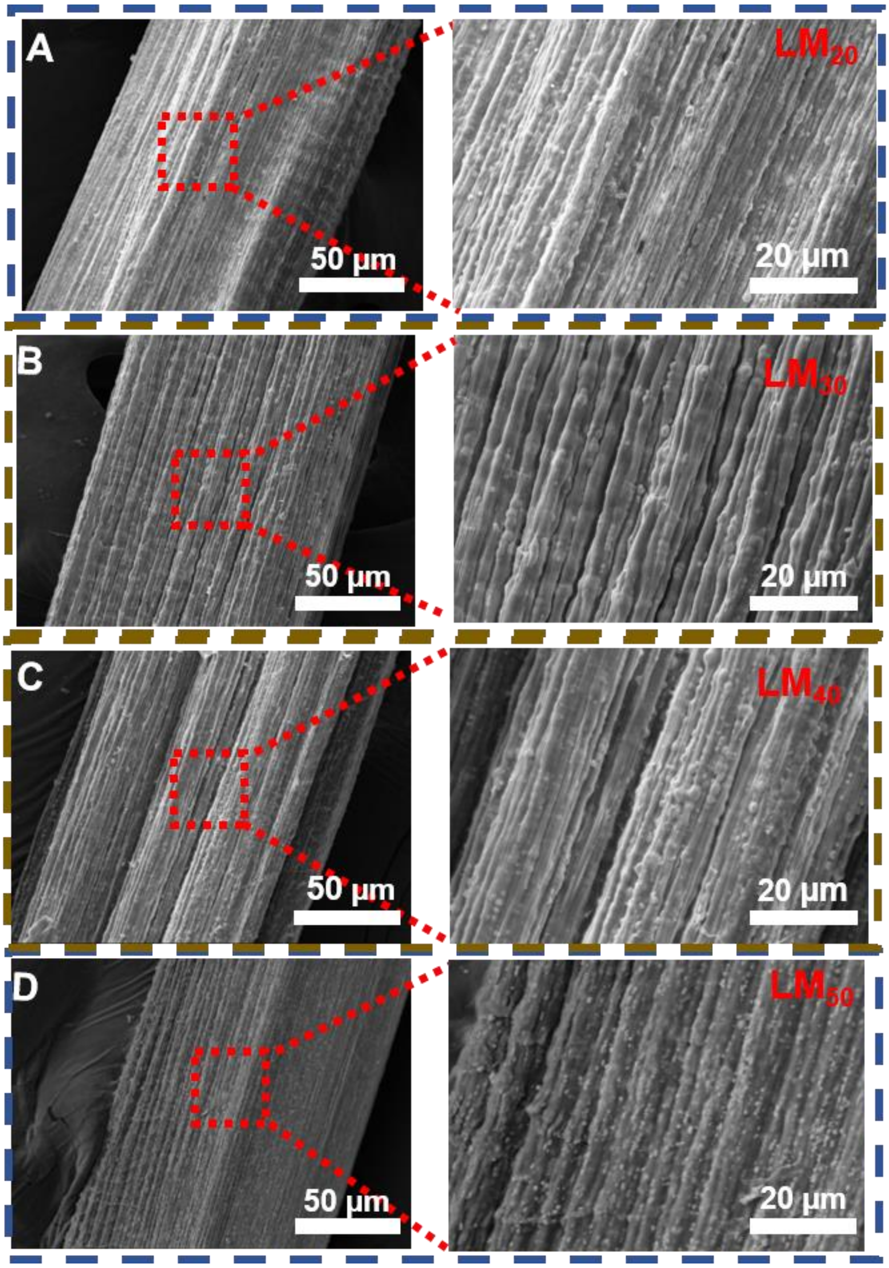

Figure S7. (A–D) SEM images of LM/alginate composite fibers with different LM content. LM content (wt%): 20 (A), 30 (B), 40 (C) and 50 (D).

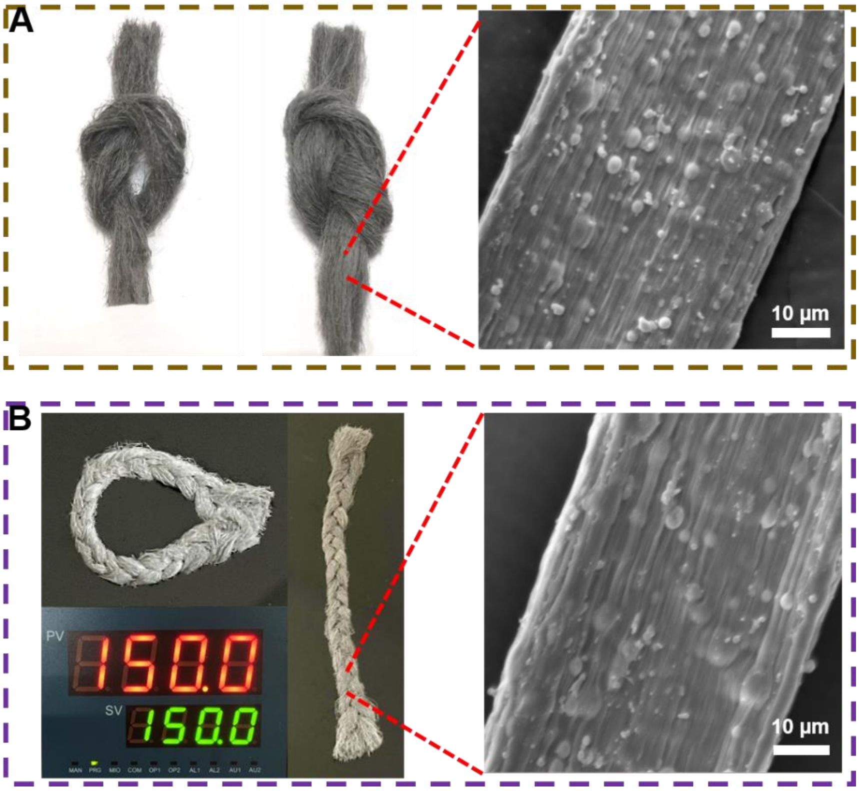

Figure S8. (A & B) The optical and SEM images of LM/alginate composite fibers immersed into the liquid nitrogen at –196 °C (A) and placed on the oven at 150 °C (B).

It can be seen that the surface of these fibers has not been destroyed.

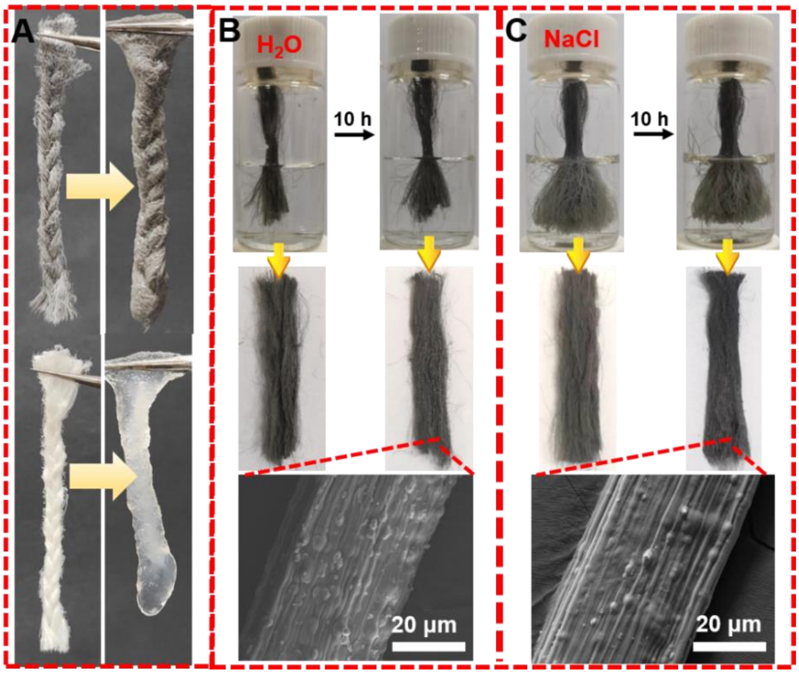

Figure S9. (A) The optical images of LM/alginate fibers and alginate fibers immersed in physiological saline solution (0.9 wt% NaCl) for 2 h. (B & C) The optical and SEM images of LM/alginate fibers immersed in pure water for 10 h (B) and 1 mol L^{-1} NaCl solution (C).

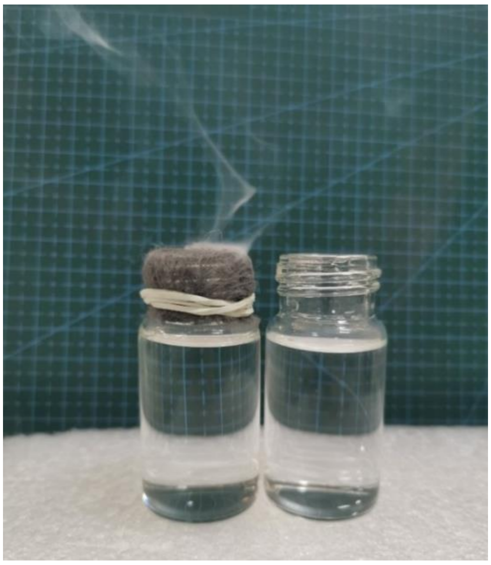

Figure S10. Gas permeability test of LM/alginate based textiles. The textiles were covered over bottles filled with hydrochloric acid, which showing the superior gas permeability.

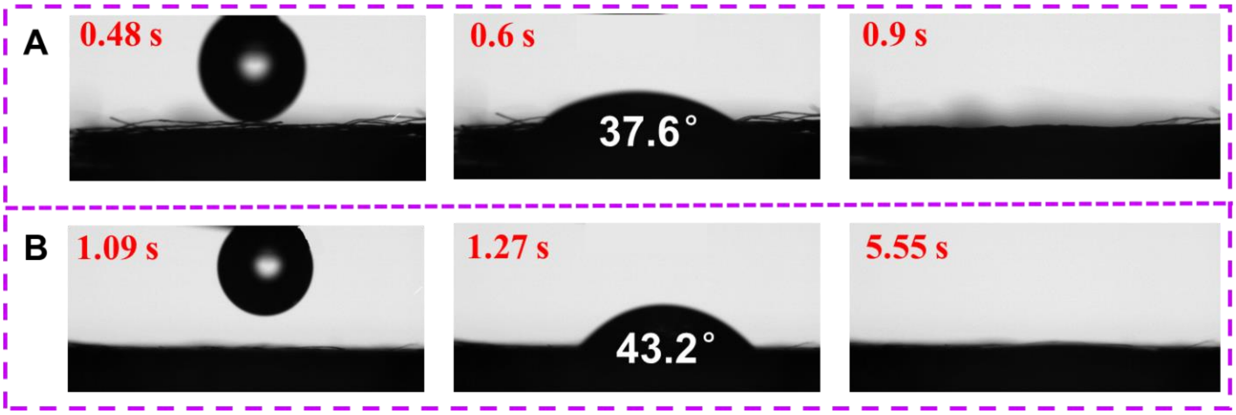

Figure S11. (A & B) The contact angle measurement of alginate fiber fabric (A) and LM/alginate composite fiber fabric (B).

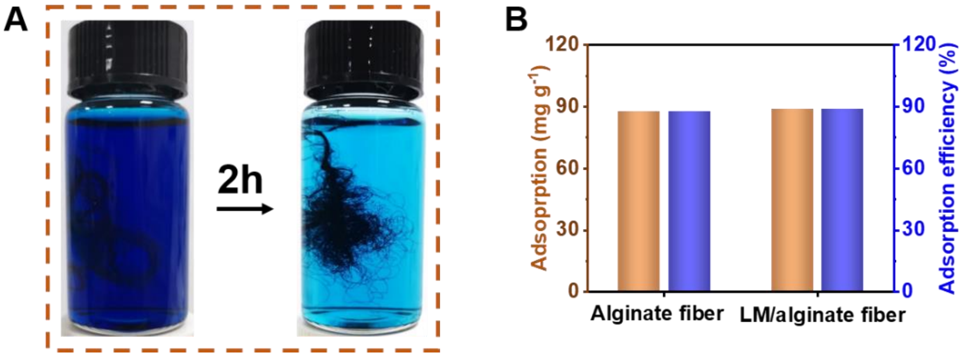

Figure S12. (A) Optical image of LM/alginate fibers (10 mg) soaked in methylene blue solution (100 mg L^{-1}). (B) Comparison of adsorption amount and adsorption efficiency of alginate fibers and LM/alginate composite fibers for methylene blue.

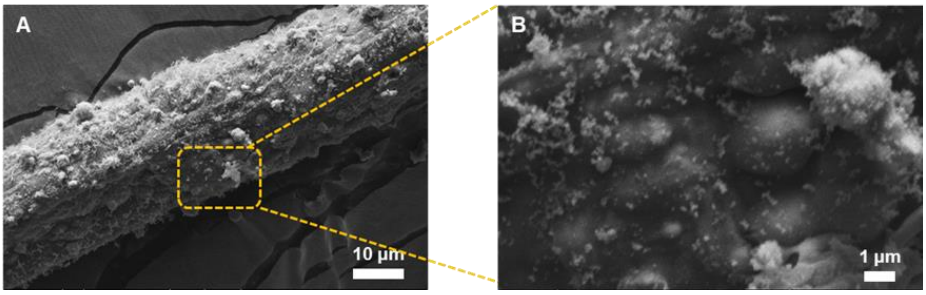

Figure S13. (A & B) SEM images of LM/alginate fiber after combustion.

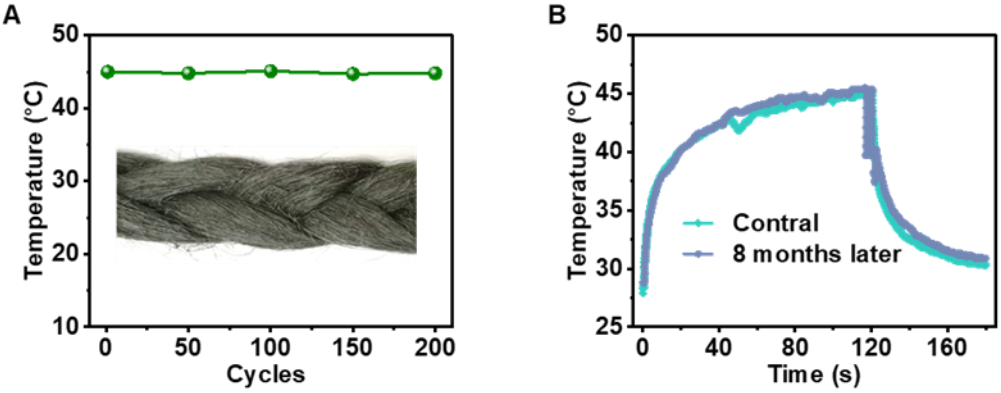

Figure S14. (A) Stability of the photo-thermal performance of the fibers for 200 cycles. (B) Temperature-time curve of LM/alginate composite fibers (ϕ_{LM}=40 wt%) under NIR irradiation. Power density of NIR: 200 mW cm^{-2}.

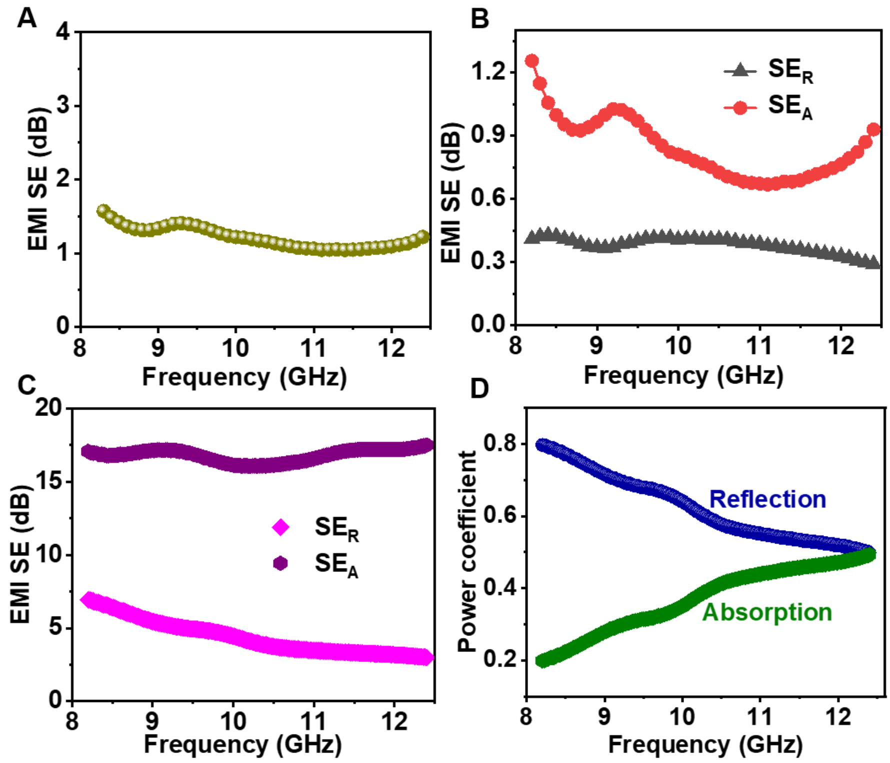

Figure S15. (A & B) EMI SE$_T$ (A), SE$_R$ and SE$_A$ (B) of alginate fibers in the frequency ranges 8.2−12.4 GHz. (C & D) SE$_R$ and SE$_A$ (C), R and A (D) of LM/alginate fibers in the frequency ranges 8.2−12.4 GHz. LM content: 50 wt%.

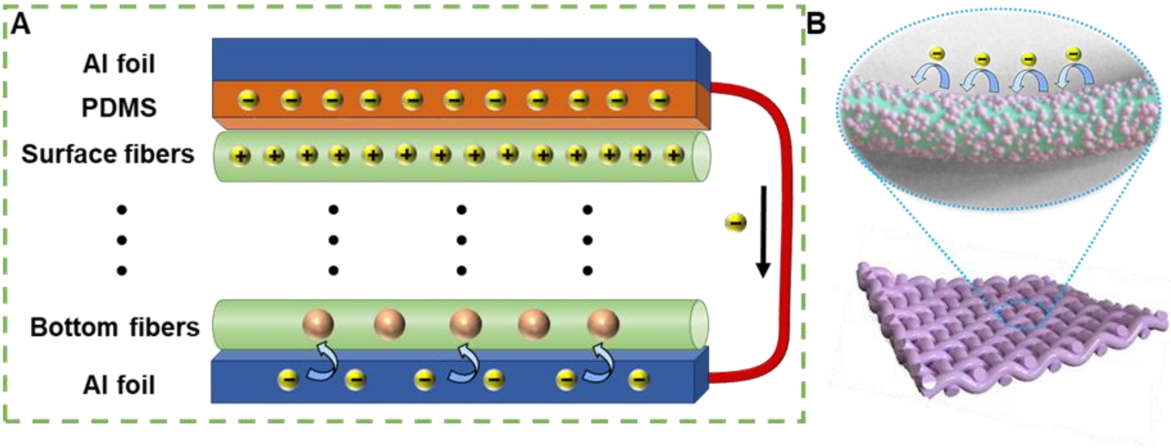

Figure S16. (A) Schematic diagram of charge trapping for LM particles. (B) Schematic diagram of electrons hopping for LM/alginate composite fibers.

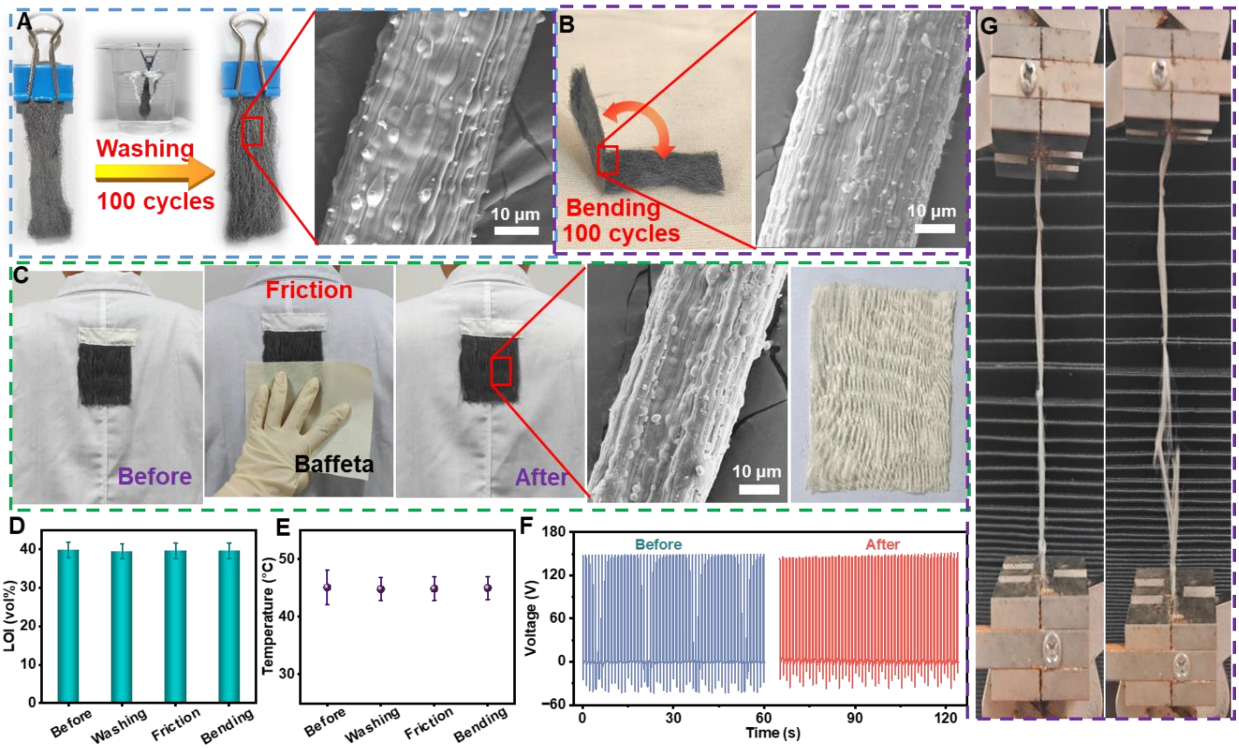

Figure S17. (A-C) Optical and SEM images before and after hundreds of cycles of LM/alginate fibers for washing test (A), bending test (B) and rubbing test (C). (D-F) LOI values (D), photo-thermal performance (E) and TENG performance (F) before and after hundreds of cycles of LM/alginate fibers for washing, rubbing and bending. (G) Mechanical test of LM/alginate fibers with three knots.

Supporting Video

Video S1. Flexibility of LM/alginate composite fibers before and after immersing into liquid nitrogen (–196 °C).

Video S2. Flame retardancy test of alginate fibers and LM/alginate fibers.
